# Maine Veterinary Medical Association

**Published:** 1895-02

**Authors:** H. H. Choate

**Affiliations:** Secretary


Secretary.

### MAINE VETERINARY MEDICAL ASSOCIATION.

THE annual meeting of the Maine Veterinary Medical Association was held at the Cony House, Augusta, January 9, 1895, at 8 P.M., Vice-President Dr. Huntington in the chair. The following members answered the roll-call: Drs. Dwinal, Barnum, Huntington, Joly, Russell, and Choate.

It was voted to present the original bill to the present Legislature, with slight changes in the amount of fines and imprisonment to be imposed for violation of the same, so as to read, as follows :

*Be it enacted by the Senate and House of Representatives in Legislature assembled*, as follows :

Section 1. No person shall advertise or hold himself out as a veterinarian or veterinary surgeon unless he is a graduate of a veterinary college or school.

Sec. 2. Any person violating the provisions of the foregoing section shall be punished by a fine of not less than five dollars nor more than fifty dollars, or by imprisonment of not more than three months, or both.

A copy of the above was ordered sent to each of the members, asking them to refer the matter to the Representative from their locality, and urge said Representative to give it his favorable consideration and support.

The Executive Committee reported favorably the names of Drs. F. M. Perry, Houlton and F. E. Freeman, Belfast, for membership in the Association.

In the absence of the chairman of the Committee on Professional Fees, Dr. Choate reported favorably the following schedule of prices, which was adopted :

| | |
|---|---|
| Single visits | $1.50 |
| More than one visit, each | 1.00 |
| Country visits per mile, one way, first mile | 1.00 |
| Country visits per mile, one way, after first mile | 0.50 |
| Night attendance | 2.00 to 5.00 |
| Per day (expenses and) | 10.00 |
| Consultation at office | 1.00 |
| Consultation with other veterinarian | 3.00 |

*Operations.*

| | |
|---|---|
| Castrating colts at operating room | $3.00 |
| Castrating colts outdoor | 5.00 |
| Castrating over 4 years | $5.00 to 10.00 |
| Castrating ridglings | 10.00 to 15.00 |
| Castrating small animals | 1.00 to 2.00 |
| Floating | 1.00 to 2.00 |
| Firing | 5.00 to 10.00 |
| Neurotomy | 5.00 to 10.00 |
| Parturition | 5.00 to 10.00 |
| Phlebotomy | 3 00 |
| Quittor | 10 00 to 15 00 |
| Removing placenta | 3.00 to 5.00 |
| Removing tumors | 5 00 to 10.00 |
| Removing molars | 3 00 to 5.00 |
| Spaying bitches | 3 00 to 5.00 |
| Tracheotomy | 3.00 to 5.00 |
| Minor operations requiring casting | 5.00 |

Drs. F. M. Perry and F. E. Freeman were elected to membership in this Association.

The election of officers resulted in the election of: *President*, George H. Bailey, D.V.S. ; *Vice-President*, F. W. Huntington, D.V.S. ; *Secretary*, H. H. Choate, D.V.S.; *Treasurer*, A. Joly, D.V.S.

Dr. Choate presented his report for the past year as Secretary, containing some suggestions, which were favorably considered by the association.

It was voted to append to the present pamphlet of the constitution and by-laws the names of the officers and members, with their addresses, also, the time for holding the meetings.

A motion was carried to have two hundred copies of the schedule of professional fees printed and distributed among the members. The motion was carried to hold the next meeting ar Belfast, April 11, 1895. The meeting then adjourned.

H. H. CHOATE,
Secretary.

It is with pleasure that I call your attention to a short report which we have prepared, and, although very incomplete, will perhaps serve to show the drift of this association during its existence. The year now closing, and

I might say, two years, which mark the period of existence of this Association, have been, on the whole, very gratifying, and, although at no special time have we been very busy, a good deal has been accomplished. About the last of September, 1892, a postal was sent to every veterinarian in the State of Maine, inviting his presence at the Preble House, Portland, for the purpose of forming a State veterinary medical association. Six veterinarians responded, and the initial meeting was held October 11th, of that year. From this time until the time for holding the October meeting in 1893 the Association progressed favorably. We have added to our list of membership at that date four new members, making in all ten. We have also presented to the State Legislature a bill to regulate the practice of veterinary medicine, which I am sorry to say was not very favorably received, and proved quite an expense to the Association. At the October meeting, 1893, the Association met with difficulties, there not being members enough present to form a quorum, and we were obliged to adjourn to meet at Lewiston, January, 1894. Here again we met with defeat, which was repeated in April. Thinking that perhaps we would have better success in Portland, we adjourned to meet there in July. This meeting lacked only one member of a quorum. During this time several schemes were employed to arouse the veterinary profession of this State from its seeming lethargy, and letter copies from the best veterinary medical journals in the country were circulated, calling attention to the importance of organization and union in this work; also, showing the great losses which must be sustained by failing to co-operate with the majority of the profession, and the great mistake in holding aloof from organizations. How far this has been successful I leave you to judge. Our meeting at Waterville, October, 1894, was a perfect success, there being the largest number of veterinarians present of any meeting, but from past experiences the association thought it best to change the number necessary for a quorum from five to three, which we hope will obviate any difficulties of this kind in the future.

At this meeting some very able articles were read and discussed, and we added to our membership three veterinarians, making the whole number to date thirteen, so that we are now represented in every city of any size in the State, except Augusta, Gardiner and Bath. This, with two applications to be acted upon at this meeting, seems very encouraging. Our by-laws, although seeming quite complete, I think would be improved by a slight addition. I would suggest that a list of officers and members with their addresses and dates of holding meetings be appended. All members should sign the constitution and by-laws, according to the import of our creed. We should then have their own signatures as proof of their good faith in joining us. In concluding this report, fellow-members, I wish to thank you all for your kind aid and support during my term of office, for the hearty good-will displayed and the many encouraging words received, all of which I fully appreciate.

H. H. CHOATE,
Secretary.

